# Sense of Relationship Entitlement of Aging Parents Toward Their Offspring (SRE-ao)—A New Concept and Measurement Tool

**DOI:** 10.3389/fpsyg.2022.885620

**Published:** 2022-06-03

**Authors:** Rami Tolmacz, Lilac Lev-Ari, Rachel Bachner-Melman, Yuval Palgi, Ehud Bodner, Darya Feldman, Ron Chakir, Boaz Ben-David

**Affiliations:** ^1^Baruch Ivcher School of Psychology, Interdisciplinary Center Herzliya, Herzliya, Israel; ^2^Clinical Psychology Graduate Program, Ruppin Academic Center, Emek Hefer, Israel; ^3^The Lior Tsfaty Center for Suicide and Mental Pain Studies, Ruppin Academic Center, Emek Hefer, Israel; ^4^School of Social Work, Hebrew University of Jerusalem, Jerusalem, Israel; ^5^Department of Gerontology, Haifa University, Haifa, Israel; ^6^Interdisciplinary Department of Social Sciences, Bar-Ilan University, Ramat Gan, Israel; ^7^Department of Speech-Language Pathology, University of Toronto, Toronto, ON, Canada; ^8^Toronto Rehabilitation Institute, University Health Networks (UHN), Toronto, ON, Canada

**Keywords:** sense of entitlement, older adults, questionnaire, parent–child relationships, mental distress, sense of loneliness, sense of belonging

## Abstract

Our sense of entitlement influences our interactions and attitudes in a range of specific relational contexts, one of them being aging parents’ relationships with their adult children. This study aimed to examine the factor structure of the Sense of Relational Entitlement—aging parents toward their offspring (SRE-ao), an 11-item questionnaire that assesses aging people’s sense of relational entitlement toward their children, and examine the associations of its subscales with related personality and mental health constructs. One thousand and six participants (24.6% men), aged 65–99, with at least one child, completed the SRE-ao, Brief Symptom Inventory, Loneliness Scale, and General Belongingness scale. The SRE-ao demonstrated good construct structure using confirmatory factor analysis. Both SRE-ao subscales (restricted and inflated sense of entitlement) were significantly and positively associated with anxiety, depression, somatization and sense of loneliness and negatively with sense of belonging. When all variables were entered into a regression model, age, anxiety, and low sense of belonging, but not sense of loneliness, positively predicted both restricted and inflated sense of entitlement. Somatization negatively predicted inflated sense of entitlement. The SRE-ao is a reliable and valid scale that can be used in clinical practice and research to enhance our understanding of parent–child relationships throughout the lifespan.

## Introduction

Our sense of entitlement, the subjective perception of what we deserve in specific situations, has recently received growing attention in psychological discourse and research (Brenner et al., 2019; Efrati et al., 2019). Sense of entitlement influences our interactions and attitudes in a wide range of relational contexts, depending on our subjective beliefs about what we deserve to receive from others. All parties to interpersonal relationships develop a sense of entitlement (Spiegel, 1987), a central determinant of how we behave in relationships and conduct our daily lives (Solomon and Leven, 1975).

Entitlement-related attitudes toward family members tend to be unique in their quality and intensity, since close family relationships evoke needs and expectations of a stronger nature than would be expected in more distant relationships (Tolmacz et al., 2016). The quality of people’s sense of entitlement toward their relatives is central to the nature of these relationships (Efrati et al., 2019). Sense of entitlement has been shown to be related to wellbeing, attachment orientations, emotional state, and life satisfaction, concepts of paramount importance in the context of family relationships (Tolmacz and Mikulincer, 2011; Tolmacz et al., 2016).

As a result of progress in medicine and public health, people live longer and enjoy better health than previously (Olshansky et al., 2012). As people age, they usually need increased help from others because of age-related health problems (Fyrand, 2010), experience difficulty adjusting to the world of technology and telecommunications and lose friends. Close family members frequently become the primary carers of aging adults (Karantzas and Simpson, 2015). Specifically, research has spotlighted children as a major source of support for their parents (Connidis and Barnett, 2018). The long-term nature and high levels of intimacy that characterize relationships between older people and their adult children are a fertile context for investigating entitlement attitudes.

The major objective of the current study was to adapt the validated “Sense of Relational Entitlement adolescents-parents” scale (SRE-ap) that measures adolescents’ expectations that their relational wishes and needs be fulfilled by their parents (Tolmacz et al., 2016) to create a scale assessing adaptive and maladaptive manifestations of older people’s sense of relational entitlement toward their children [Sense of Relational Entitlement—older adults toward children (SRE-ao)]. We also sought to examine the scale’s factor structure and the associations of its subscales with personality and emotion-related variables.

### Developmental Perspectives on Entitlement

Sense of relational entitlement has traditionally been perceived as pathological, for example as a criterion for narcissistic personality disorder (American Psychiatric Association, 2000) or psychopathy (Hare, 1999). However, this concept has expanded to include the healthy assertion of needs and rights (Levin, 1970; Kriegman, 1983; Moses and Moses-Hrushovski, 1990). Wolfe and Bailey (2003) and Tolmacz (2011) have embedded a sense of relational entitlement within the developmental perspective of attachment theory. According to this reconceptualization, a sense of entitlement develops largely in the context of attachment relationships with primary caregivers. Three different types of entitlement patterns, including both pathological and healthy aspects of needs and rights, are thought to form within our internal working models: An *assertive* sense of entitlement allows the formation of realistic expectations from others. An *inflated* sense of entitlement leads to unrealistic expectations that all our need and wishes be fulfilled. A *restricted* sense of entitlement involves ignoring rather than expressing authentic needs and wishes. Even though they offer protection from psychic pain (Moses and Moses-Hrushovski, 1990), both inflated and restricted relational entitlement may be expressions of impaired self-esteem that lead to frustrating interactions.

In line with view Bowlby’s (1969, 1982) that the attachment system influences us “from the cradle to the grave” (p. 208), attachment theory can be seen as a conceptual framework for adults’ support of their aging parents (Steele et al., 2004). Similarly, conceptualizing sense of entitlement in terms of attachment theory frames it as a universal phenomenon and stresses its critical role throughout the lifespan. Previous research has shown the importance of attitudes toward entitlement during childhood and adolescence, when children rely on their parental figures as a secure base (Tolmacz and Mikulincer, 2011), and during adulthood, in the context of romantic relationships (Feeney, 2004; Brenner et al., 2019). The implications of relational entitlement have yet to be investigated in relation to older adults’ connectedness. In this study, we explored sense of relational entitlement in the context of aging parents’ relationships with their adult children.

### Measuring Sense of Relational Entitlement

In the past, inflated sense of entitlement was considered an expression of narcissism (Emmons, 1984). However, it became clear that the Narcissistic Personality Inventory entitlement subscale (NPI, Raskin and Hall, 1979) was an inadequate measure of entitlement. Specifically, its items failed to load onto a single, differentiated factor (Emmons, 1984), lacked face validity, had low reliability and were too few in number (Campbell et al., 2004). The distinction between narcissism and an inflated sense of entitlement is also supported theoretically. Whereas, narcissistic attitudes usually imply a grandiose sense of self, Freud pointed out as early as 1916 that entitlement demands are often based on a sense of injustice stemming, for example, from a history of congenital diseases or disabilities (Freud, 1916). This led to the development of two independent scales assessing a general sense of entitlement, the Entitlement Attitude Scale (Nadkarni and Malone, 1989), and the Psychological Entitlement Scale (Campbell et al., 2004). It has nevertheless been stressed that sense of entitlement is situation- or relation-specific, for example to relationships between adolescents and their parents (Tolmacz et al., 2016), with subjective meanings attributed to this context (Kriegman, 1983; Moses and Moses-Hrushovski, 1990).

Tolmacz and Mikulincer (2011) developed and validated the 33-item SRE scale to measure sense of entitlement in adult romantic relationships. They concluded that sense of entitlement in close relationships should be conceived of and measured differently from narcissism and general sense of entitlement. A series of factor analyses pointed to three main factors, inflated, assertive, and restricted sense of entitlement. Scores on the inflated and restricted subscales were positively associated with emotional difficulties and attachment insecurities and negatively with adaptive personality dispositions and wellbeing. Conversely, scores on the assertive subscale were associated with more positive personality dispositions (Tolmacz and Mikulincer, 2011). More recently, research on entitlement has indicated its importance among adolescents (Tolmacz et al., 2016). An investigation of adolescents’ sense of entitlement from their parents found that an imbalanced sense of entitlement (restricted or exaggerated) was associated with emotional problems, lower wellbeing, less positive mood and life satisfaction, and more attachment insecurities (Tolmacz et al., 2016). Recently, due to certain drawbacks of the SRE, particularly the assertive subscale, Tolmacz et al. (2021) have recommended measuring sense of relational entitlement using an approach parallel to the measurement of attachment orientation with the Experiences in Close Relationships scale (ECR; Brennan et al., 1998). Just as healthy attachment is indicated by low scores on both ECR dimensions, anxiety and avoidance, a healthy sense of entitlement is indicated by low scores on the two imbalanced entitlement dimensions, inflation and restriction.

### The Current Study

The main goal of the present study was to develop a self-report scale, the SRE-ao, to assess the extent to which older adults expect their children to fulfill their needs and wishes. We also aimed to tap aging people’s affective and cognitive responses when their needs and wishes are *not* fulfilled by their children. The SRE-ao scale was based on the SRE-ap scale (Tolmacz et al., 2016), and we examined whether its factor structure is replicated. The hypothesized subscales were *inflated* sense of relational entitlement, that is aging parent’s belief that their children should meet all their needs and wishes and *restricted* sense of relational entitlement, or the reluctance of older people to convey their wishes and needs to their children because they doubt their legitimacy.

In order to examine the construct validity of the SRE-ao, we examined the correlations between its subscales and related personality and mental health constructs. Specifically, we tested the hypothesis that aging parents’ sense of relational entitlement toward their children would be associated with emotional difficulties. Depression and anxiety are common among older people (Byers et al., 2010) and affect their quality of life (Atkins et al., 2013) and physical health (Blazer, 2009). Moreover, their mental health can be strongly affected by the quality of their relationships with their adult children. Pertinently, positive relationships were found to be protective of depressive symptoms, whereas conflictual relationships were found to be related to emotional distress (Reczek and Zhang, 2016). Moreover, sense of entitlement in adults has been linked to both depression (McGinn et al., 2005; Halvorsen et al., 2010) and anxiety (Muris, 2006; Tolmacz and Mikulincer, 2011). Grubbs and Exline (2016) concluded that inflated entitlement confers vulnerability to psychological distress. We therefore hypothesized that both SRE-ao subscales would be significantly and positively associated with anxiety and depressive symptoms.

We also examined the association between older adults’ sense of relational entitlement toward their offspring and two measures related to social relationships, sense of loneliness and sense of belonging. Old people experience a decrease in social connection, causing emotional and social loneliness (Hawkley et al., 2010), which may jeopardize wellbeing (Aylaz et al., 2012) and self-esteem (Dahlberg and McKee, 2014). The association between loneliness and depression is well-documented, particularly in older adults (Domènech-Abella et al., 2017). A sense of belonging, however, is protective against loneliness (Prieto-Flores et al., 2011) and has been found to be negatively associated with depression and suicide in aging populations (Vanderhorst and McLaren, 2005) and positively with wellbeing (Park, 2009). Loneliness has been defined as a discrepancy between desired and real social relations (Peplau and Perlman, 1982), so inevitably evokes issues of entitlement. Moreover, wellbeing, depression, and self-esteem, all associated with loneliness, have also been linked to sense of entitlement. We therefore hypothesized that an impaired sense of relational entitlement (inflated and restricted) would be positively associated with a sense of loneliness and negatively with a sense of belonging.

We administered the SRE-ao to a large community sample of older adults who were parents. We hypothesized that:

1. *The SRE-ao will demonstrate good construct structure [using Confirmatory Factor Analysis (CFA)].*
2. *The inflated and restricted subscales of the SRE-ao would be significantly and positively associated with each of the anxiety, depression, and somatization subscales of the Brief Symptoms Inventory (BSI).*
3. *The inflated and restricted subscales of the SRE-ao would be significantly and positively associated with a sense of loneliness and negatively with a sense of belonging.*

*Mental distress (BSI anxiety, depression, and somatization symptoms) and relationship indices (sense of belonging and sense of loneliness) would be significantly associated with inflated and restricted sense of relational entitlement (SRE-ao), when controlling for mental distress, age, and gender.*

## Method

### Participants

A total of 1,006 participants aged 65–99 years (*M* = 73.37, *SD* = 7.29) participated in the study (24.6% men). Inclusion criteria included age above 65 and at least one child above the age of 21.

Ten (1%) of the participants were single, 623 (61.9%) were married, 211 (20.9%) were widows, and 162 (16.1%) were divorced. Most (84%) lived in their own home, 13% in an assisted living community, 2% with their children and 1% reported another living arrangement. All participants were Jewish. Most (*n* = 797, 79.2%) described themselves as secular, 173 (17.2%) as traditional, and 36 (3.58%) as religious.

### Measures

#### Sense of Relational Entitlement

The SRE-ao was used to assess the degree to which the participants expected their adult children to fulfill their needs. Its two factors include items that tap two facets of relational entitlement, exaggerated and restricted sense of entitlement. The original questionnaire had 15 items. Item 12 was deleted because it was not relevant to the aged (when my parents frustrate me, I sometimes think of running away from home) and another three (1, 8, and 15) because of low factor loadings. Eleven items were adapted from the restricted and inflated subscales of the SRE-ap (Tolmacz et al., 2016). The assertive subscale was not included because of its poor validity in previous studies (e.g., Tolmacz and Mikulincer, 2011; Tolmacz et al., 2016). Other references to parents were altered to references to children. For example, “Sometimes, I think my parents love me more than I deserve” was changed to: “Sometimes, I think my children love me more than I deserve.” Items were reviewed by four clinical psycho-gerontologists. Responses were noted on a 5-point scale between 1 (very inaccurate) and 5 (very accurate). To ensure that items and instructions were clear to the target population, a preliminary version of the scale was administered to a sample of 10 older adults, who were interviewed about the clarity of the questionnaire. Cronbach’s alpha for the restricted subscale in this study was 0.73 and for the inflated subscale 0.69.

#### Mental Distress

The Brief Symptom Inventory-18 (BSI-18; Derogatis, 2001) was used to assess aspects of mental distress during the past week. The scale consists of three subscales: somatization (e.g., “numbness or tingling in parts of your body”), depression (e.g., “feeling hopeless about the future”), and anxiety (e.g., “feeling tense or keyed up”). Responses range from 1 (not at all) to 5 (to an extreme). Subscale means and a global mean are calculated, with high scores indicating high distress. Reliability has been found to be good for somatization (α = 0.74) and anxiety α = 0.79) and very good for depression (α = 0.84) and the global score (α = 0.89; Recklitis et al., 2006). In this study, alpha Cronbach was 0.90 for the whole questionnaire, 0.87 for depression, 0.74 for somatization, and 0.77 for anxiety.

#### Sense of Loneliness

Loneliness was measured by the 6-item de Loneliness Scale (LS; De Jong Gierveld and Van Tilburg, 2006). The questionnaire includes two subscales, emotional loneliness, or lack of close relationships (e.g., “There are enough people I feel close to”; reversed) and social loneliness, or lack of social network (e.g., “there are many people I can trust completely”; reversed). Responses range from 1 (all the time) to 5 (none of the time). The overall LS score was calculated by summing the two subscales. The LS generally has good reliability (α = 0.70; De Jong Gierveld and Van Tilburg, 2006); in the current study, alpha was 0.71.

#### Sense of Belonging

The General Belongingness Scale (GBS; Malone et al., 2012) was used to assess sense of belonging. The scale consists of 12 items divided into two subscales: acceptance/inclusion (e.g., “When I am with other people, I feel included”) and rejection/exclusion (e.g., “I feel like an outsider”). Responses range from 1 (totally object) to 5 (totally agree). Scores were the mean of all items after reversing the rejection/exclusion items. The scale has excellent reliability (Cronbach’s alpha = 0.92), and in the current study, it was very good (α = 0.83).

### Procedure

Six research assistants underwent professional training, administered the questionnaires via pen and paper in assisted living facilities, at community centers and via snowball sampling. Participants were asked to contact and refer friends willing to participate. In some cases, questionnaires were distributed after a lecture about mindfulness offered by one of the research assistants. No monetary reward was offered in exchange for participation. Informed consent was obtained as required by the Institutional Review Board of the institution of the first author.

### Data Analyses

Structural equation analysis using AMOS 23.0 was conducted for the CFA. The following values were set as criteria for the acceptance of the model: Comparative Fit Index (CFI) > 0.90 (Bentler and Bonett, 1980), root-mean-square error of approximation (RMSEA) < 0.08 (Browne and Cudeck, 1993), and SRMR < 0.08. All other analyses were conducted using the Statistical Package for the Social Sciences (SPSS, version 23). To test for convergent validity, Pearson correlations were calculated between SRE-eo subscale scores and other indices.

## Results

*Hypothesis 1: The SRE-ao will demonstrate good construct structure (using CFA).*

### Confirmatory Factor Analysis for the Sense of Relational Entitlement—Aging Parents Toward Their Offspring (*N* = 1,006)

**FIGURE 1:**
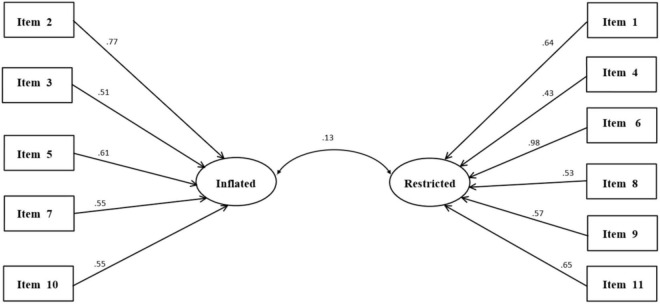
CFA of the two-factor model for the SRE-ao. All paths were statistically significant at *p* < 0.001. Latent variables are shown by ellipses and observed variables by rectangles. Arrows between latent variables indicate correlations between latent variables. Correlations between latent and observed variables were significant at *p* < 0.001.

The consistency of the data with the constructs (factors) inflated and restricted sense of relational entitlement was examined using CFA. The model showed good fit for the data [χ^2^_(34)_ = 117.70; *p* < 0.001; CFI = 0.96, RMSEA = 0.05; SRMR = 0.04] (see Figure 1). Cronbach’s alphas were 0.69 for inflated sense of relational entitlement and 0.73 for restricted sense of relational entitlement.

*Hypothesis 2: The inflated and restricted subscales of the SRE-ao would be significantly and positively associated with BSI-18 anxiety, depression, and somatization.*

Before examining this hypothesis, we assessed associations between all indices and age. No correlations were statistically significant. Pearson correlations between the SRE-ao subscale scores and BSI-18 anxiety, depression, and somatization are presented in Table 1. Restricted and inflated sense of relational entitlement were significantly and positively correlated. SRE-ao inflated and restricted sense of entitlement was significantly and positively associated with BSI-18 symptoms of depression, anxiety, and somatization.

**TABLE 1:** Correlations between SRE-ao subscales, BSI-18 subscales, GBS, and LS.

	Inflated SRE-ao	Restricted SRE-ao	Mean (SD)
Inflated SRE-ao			2.03 (0.67)
Restricted SRE-ao	0.29		1.75 (0.75)
BSI-18 depression	0.18	0.19	1.78 (0.86)
BSI-18 anxiety	0.26	0.20	1.67 (0.63)
BSI-18 somatization	0.10	0.16	1.58 (0.66)
GBS sense of belongingness	−0.06	−0.32***	3.85 (0.73)
LS sense of loneliness	0.21**	0.17*	2.13 (1.57)

*All correlations were significant at the p < 0.001 (2-tailed). Inflated SRE-ao, Inflated Sense of Relational Entitlement—aging parents toward their offspring; Restricted SRE-ao, Restricted Sense of Relational Entitlement—aging parents toward their offspring; BSI-18, Brief Symptoms Inventory-18; GBS, General Belongingness Scale; LS, Loneliness Scale. Correlations between SRE-ao subscales and BSI-18 subscales N = 1,006. For the correlations between GBS, LS, and SRE-ao subscales N = 197.*

*Hypothesis 3: Inflated and restricted subscales of the SRE-ao would be significantly and positively associated with a sense of loneliness (LS) and negatively with a sense of belonging (GBS).*

Pearson correlations between the SRE-ao subscale scores and sense of loneliness (LS) and sense of belongingness (GBS) are presented in Table 1. Restricted sense of relational entitlement (SRE-ao) was significantly and negatively correlated with sense of belonging (GBS), and restricted and inflated sense of entitlement (SRE-ao) were significantly and positively correlated with sense of loneliness (LS).

*Mental distress (BSI-18 anxiety, depression, and somatization symptoms) and relationship indices [sense of belongingness (GBS) and sense of loneliness (LS)] would be significantly associated with inflated and restricted sense of relational entitlement (SRE-ao) when controlling for mental distress, age, and gender.*

To test these hypotheses, we conducted two hierarchical regression analyses with inflated and restricted sense of entitlement (SRE-ao) as dependent variables (see Table 2). Age was entered into the model in the first step, BSI-18 subscales were added in the second, and relational indices in the third.

**TABLE 2:** Prediction of inflated and restricted subscales of the SRE-ao by BSI-18 anxiety, depression, and somatization subscales, sense of belonging (GBS), and sense of loneliness (LS), when controlling for age and gender (*n* = 197).

Predictor	Inflated SRE-ao	Restricted SRE-ao
**Step 1**		
R^2^/Adj. R^2/^F_(_*_*df*_*_)_	0.01/0.001/_(2, 194)_ = 0.95	0.06/0.05/_(2, 194)_ = 5.63**
Age	0.06	0.21**
Gender (0 = male)	–0.07	–0.07
**Step 2**		
R^2^/Adj. R^2/^ΔR/F_(_*_*df*_*_)_	0.13/0.10/0.12***/_(5, 191)_ = 5.44***	0.18/0.16/0.12***/_(5, 191)_ = 8.19***
Age	0.09	0.20**
Gender (0 = male)	–0.11	–0.08
BSI-18 somatization	−0.36**	0.10
BSI-18 depression	0.11	0.02
BSI-18 anxiety	0.37***	0.28***
**Step 3**		
R^2^/Adj. R^2/^ΔR/F_(_*_*df*_*_)_	0.16/0.13/0.04*/_(7, 189)_ = 5.29***	0.22/0.19/0.04**/_(7, 189)_ = 7.52***
Age	0.08	0.19**
Gender (0 = male)	–0.10	–0.09
BSI-18 somatization	−0.41**	0.04
BSI-18 depression	–0.05	–0.21
BSI-18 anxiety	0.33***	0.32***
Sense of belonging (GBS)	–0.22	−0.36**
Sense of loneliness (LS)	0.13	–0.91

p < 0.01*, p < 0.05**, p < 0.001***. Inflated SRE-ao, Inflated Sense of Relational Entitlement—elderly for children; Restricted SRE-ao, Restricted Sense of Entitlement—aging adults toward their offspring; BSI-18, Brief Symptom Inventory-18; GBS, General Belongingness Scale; LS, Loneliness Scale.

As can be seen from Table 2, age predicted restricted but not inflated sense of relational entitlement (SRE-ao). BMI-18 somatization symptoms negatively predicted, and BSI-18 anxiety positively predicted SRE-ao inflated sense of relational entitlement, adding 11% to the explained variance. Adding sense of belonging (GBS) and loneliness (LS) to the model did not enhance it. The older the participants and the more anxiety symptoms they reported, the more inflated was their sense of relational entitlement. The more somatization symptoms participants reported, the less inflated was their sense of relational entitlement.

SRE-ao restricted sense of relational entitlement was positively predicted by age and BSI-18 anxiety, which predicted 15% of the explained variance. Sense of belonging (GBS) also negatively predicted SRE-ao restricted sense of relational entitlement, adding another 4% to the explained variance. The older and the more anxious participants were, the more restricted was their sense of relational entitlement. The greater their sense of belonging, the less restricted was their sense of relational entitlement.

The results of the regression analysis led us to believe that emotional distress (BSI anxiety, depression, and somatization) may mediate the relationship between sense of loneliness (LS) and sense of relational entitlement (SRE-ac). To test this *post-hoc* hypothesis, we conducted two mediation analyses using Process (Hayes, 2012) with sense of loneliness (LS) as the independent variable and BSI anxiety, depression, and somatization as the mediating variables. The dependent variable was restricted sense of entitlement in the first analysis and inflated sense of entitlement in the second (SRE-ac). BSI anxiety fully mediated the relationship between sense of loneliness (LS) and restricted sense of entitlement (SRE-ac) (LLCI = 0.10; ULCI = 0.47). Anxiety (LLCI = 0.13; ULCI = 0.48) and somatization (LLCI = −0.47; ULCI = −0.07) partially mediated the relationship between sense of loneliness (LS) and inflated sense of entitlement (SRE-ac).

## Discussion

As people age, they usually need increased assistance and their children frequently become their primary carers. Therefore, the relationship of aging parents with their offspring is of primary importance, and the sense of entitlement among older adults toward their children is a basic component in the dynamics of this relationship. The self-report instrument we propose (SRE-ao) is specifically designed to assess this sense of entitlement in clinical practice and in research. In this study, we administered the SRE-ao to a sample of over 1,000 old people with adult children in Israel and provide initial evidence for its validity and factor structure.

The SRE-ao scale (Appendix 1) is an 11-item self-report questionnaire adapted from the SRE-ap scale (Tolmacz et al., 2016; Appendix 2) that assesses the degree to which aging parents expect their adult offspring to attend to and fulfill their needs and wishes, and their affective and cognitive response when their children fail to do this. The results of this study expand previous research on the sense of relational entitlement in romantic relationships (Tolmacz and Mikulincer, 2011; George-Levi et al., 2014) and in adolescent-parent relationships (Tolmacz et al., 2016), and support the claim “that the same underlying mechanisms engender feelings of entitlement in different intimate relationships across the life span” (Tolmacz et al., 2016; p. 136). Findings are also congruous with recent evidence suggesting that attachment mechanisms and triggers have similar effects on young and older adults (Nagar et al., in press) and that emotional and social intelligence are preserved skills in aging, even in dementia (Berenbaum et al., 2020).

The SRE-ao was found to have good psychometric properties and CFA indicated that it retains the two-factor model of the SRE-ap scale. Its two subscales are *inflated sense of entitlement*, the belief of aging people that their offspring should meet all their needs and wishes, and *restricted sense of entitlement*, aging parents attitude of reluctance to convey their wishes and needs to their offspring because they do not experience them as valid. These subscales demonstrated good internal reliability and were significantly and positively associated. The SRE-ao also showed good convergent validity. Both inflated and restricted sense of entitlement were positively and significantly associated with symptoms of depression, anxiety, somatization, and a sense of loneliness. Restricted sense of entitlement was associated negatively and significantly with sense of belonging. These findings support the construct validity of the questionnaire.

As hypothesized, symptoms of anxiety, depression, and somatization were positively and significantly associated with both inflated and restricted sense of relational entitlement. This suggests that older adults who experience imbalance in their sense of entitlement i.e., feel either over- or under-entitled to the fulfillment of their relational needs, are prone to experience higher levels of mental distress. This is in line with previous findings on the maladaptive nature of both restricted and inflated forms of entitlement. Previous research has shown that an imbalanced sense of relational entitlement is significantly associated with negative mood, emotional distress, and low levels of wellbeing and life satisfaction in adults (Halvorsen et al., 2010; George-Levi et al., 2014; Grubbs and Exline, 2016) and with increased risk for emotional problems in the context of couple relationships (Tolmacz and Mikulincer, 2011). Tolmacz et al. (2016) also found that, among adolescents, both forms of entitlement are associated with risk for depression, anxiety disorders, and school avoidance, and with low levels of positive mood, self-esteem, and life satisfaction.

A cross-sectional study, of course, does not allow us to infer causality. An imbalanced sense of relational entitlement in aging parents toward their adult children may generate mental stress by leading to interpersonal difficulties in ongoing interactions between aging parents and their adult children. Alternatively, emotional problems may emerge first and lead to maladaptive expressions of entitlement. A restricted or inflated sense of relational entitlement may serve as a coping strategy, lowering subjectively illegitimate expectations of need fulfillment from adult children or expressing exaggerated demands and expectations from them, to express or alleviate distress. It seems likely that in old age, mental distress interacts with an imbalanced sense of entitlement in a vicious cycle, with distress engendering an imbalance in the expression of needs and expectations from children, and frustration and disappointment when needs are not satisfied in turn engendering distress. Future studies would do well to use a cross-lagged design assessing both types of entitlement and different forms of mental distress at two time points.

As hypothesized, sense of loneliness was linked to both forms of imbalanced entitlement in this sample of aging people. The connection with inflated sense of entitlement may be explained by their tendency to shy away from close contact with their adult children for fear of being hurt when their needs are frustrated, setting the scene for feelings of loneliness. The connection with restricted sense of entitlement is hardly surprising and reflects the tendency of certain individuals to lower or relinquish expectations from their children, which may eventually lead them to experience a sense of loneliness. Similarly, aging parents with a restricted sense of entitlement vis-à-vis their children may tend to avoid expressing their needs from them and consequently feel isolated and lonely.

In addition, restricted sense of entitlement was significantly and negatively associated with sense of belonging in our sample. This finding seems intuitive, since most people tend to avoid expressing interpersonal needs when they feel they do not really belong socially, in this context within their family unit. This idea is supported by clinical evidence and theoretical conceptualizations linking a sense of under-entitlement with impaired self-esteem (e.g., Levin, 1970; Kriegman, 1983; Moses and Moses-Hrushovski, 1990), which is in turn associated with a sense of loneliness in older adults (Dahlberg and McKee, 2014).

When all study variables were entered into a regression model predicting imbalanced sense of entitlement, anxiety was a significant, positive predictor of both restricted and inflated sense of entitlement. Interpersonal needs may become more pronounced with a rise in anxiety, intensifying feelings relevant to entitlement. Age and a low sense of belonging also strongly predicted restricted sense of entitlement in aging parents. As people with a restricted sense of entitlement age, the reasons that lead them to ignore and not express authentic needs and wishes in the first place may intensify and further reduce their sense of entitlement. In older adults, a low sense of belonging is associated with depression and suicidal ideation (e.g., Vanderhorst and McLaren, 2005) and a high sense of belonging to reasons for living (Kissane and McLaren, 2006). This study underscores the importance of a sense of belonging for older adults’ willingness to openly communicate their needs to their offspring rather than stifling or exaggerating them.

Surprisingly, loneliness predicted neither inflated nor restricted sense of entitlement. Since loneliness and anxiety are closely linked in among older adults (Khademi et al., 2015) and anxiety significantly predicted an imbalanced sense of entitlement, it seemed feasible that the anxiety connected to loneliness, rather than loneliness *per se*, may have driven the prediction of imbalanced relational entitlement. This hypothesis was confirmed by *post-hoc* mediation analyses that showed that anxiety fully explained the connection of loneliness to inflated entitlement and that anxiety and somatization partially explained its connection to a restricted entitlement.

Interestingly, when all study variables were entered into a regression model predicting imbalanced sense of entitlement, somatization was *negatively* correlated with inflated sense of entitlement. This surprising finding adds to those of Li et al. (2018), who found that somatization was positively associated with grit and social support. One possible explanation may be that in the regression model, age, and gender were controlled for. In aging parents, there may be a difference between younger and older people. This may also have something to do with real physical difficulties. For older, more frail people, there may be a positive association between somatization or real physical ailment and inflated sense of entitlement, whereas for younger, healthier people, inflated sense of entitlement (that may still be congruent with actual life events) may be protective against somatization. Another possible explanation is that this correlational reversal reflects a confounding effect of BSI depression and anxiety. Other factors that influence the relationship between somatization and SRE-ac should be identified in future research. Future studies would also do well to elucidate the role of somatization and similar variables in predicting a sense of relational entitlement in aging adults and assess physical complaints and age as co-variables.

Relationships between elderly parents and their adult children are important and worthy of research attention, and the SRE-ac stands to expand our understanding of factors that influence this relationship. In addition, previous studies have indicated that sense of relational entitlement in younger populations is strongly connected to personality variables such as self-esteem, attachment orientations, and wellbeing (Tolmacz and Mikulincer, 2011; Tolmacz et al., 2022). Future studies should examine whether or not this holds true for older populations as well. These lines of research could lead to the development of interventions on the individual and intergenerational levels that can help adults to improve various aspects of their relationships with their parents as they age.

This study has several limitations. First, the data collected was based on self-report rather than real-life interactions and are therefore susceptible to self-serving biases. Second, items in the SRE-ao refer generically to “my children” and do therefore not tap any differences in sense of entitlement that older parents may hold toward different sons/daughters. Third, the study was cross sectional, so that no conclusions can be drawn about temporal development and effects of the study variables. Finally, most participants were female, married, lived in their own homes, and completed questionnaires online. Results may not be generalizable to older men, to people who have lost their spouse, who live in assisted living facilities, and/or who have few computer skills.

In conclusion, this study proposes an 11-item self-report scale (SRE-ao) that assesses sense of entitlement in aging parents toward their adult children and provides initial evidence for its validity and two-factor structure. The SRE-ao is associated with depression, anxiety, somatization, and loneliness and adds to a series of questionnaires assessing restricted and inflated sense of entitlement in specific kinds of relationships. We hope that the SRE-ao will be used in clinical practice and in research to expand our understanding of parent–child relationships throughout the lifespan.

## Data Availability Statement

The raw data supporting the conclusions of this article will be made available by the authors, without undue reservation.

## Ethics Statement

The studies involving human participants were reviewed and approved by Reichman University Ivcher School of Psychology. The patients/participants provided their written informed consent to participate in this study.

## Author Contributions

RT, LL-A, RB-M, YP, EB, and BB-D contributed to the conception and design of the study. DF and RC organized the database. LL-A, DF, and RC performed the statistical analysis. RT, LL-A, and RB-M wrote the first draft of the manuscript. LL-A wrote sections of the manuscript. All authors contributed to manuscript revision, read, and approved the submitted version.

## Conflict of Interest

The authors declare that the research was conducted in the absence of any commercial or financial relationships that could be construed as a potential conflict of interest.

## Publisher’s Note

All claims expressed in this article are solely those of the authors and do not necessarily represent those of their affiliated organizations, or those of the publisher, the editors and the reviewers. Any product that may be evaluated in this article, or claim that may be made by its manufacturer, is not guaranteed or endorsed by the publisher.

## Appendix 1 | SRE-ao

**Table:** The following statements concern attitudes, feelings, beliefs, and reactions in your relationships with your children. Please respond to each statement by indicating how much you agree or disagree with it.

1	2	3		4		5

Strongly disagree	Disagree	Neutral/Mixed		Agree		Strongly agree
1	I spend a lot of time thinking about my children’s weaknesses		1		2	3
2	When my children hurt me, I immediately feel I cannot trust them		1		2	3
3	I often feel my children deserve a better parent than me		1		2	3
4	Sometimes I have a lot of criticisms of my children		1		2	3
5	When my children frustrate me, I usually feel I do not deserve it		1		2	3
6	I often asking myself if I deserve such good children that I have		1		2	3
7	I feel that I get from my children more than I deserve		1		2	3
8	I feel my children deserve more than they get from me		1		2	3
9	Sometimes I think my children love me more than I deserve		1		2	3
10	Sometimes I feel I’m not good enough for my children		1		2	3
11	Usually, when my children frustrate me, I am extremely angry		1		2	3

*Inflated sense of entitlement 1, 2, 4, 5, 11.*

*Restricted sense of entitlement 3, 6–10.*

## Appendix 2 - SRE-ap

**Table:** The following statements concern attitudes, feelings, beliefs, and reactions in your relationships with your parent. Please respond to each statement by indicating how much you agree or disagree with it.

1		2	3		4			5	

Strongly disagree		Disagree	Neutral/Mixed		Agree			Strongly agree	
1	When my parents make me angry, I sometimes regret the fact that I don’t have other parents			1		2	3	4	5
2	I spend a lot of time thinking about my parents’ weaknesses			1		2	3	4	5
3	When my parents hurt me, I immediately feel I cannot trust them			1		2	3	4	5
4	I often feel my parents deserve a better child than me			1		2	3	4	5
5	Sometimes I have a lot of criticisms of my parents			1		2	3	4	5
6	When my parents frustrate me, I get very angry			1		2	3	4	5
7	When my parents frustrate me, I feel I do not deserve it			1		2	3	4	5
8	Usually, when my parents compliment me, I believe I deserve it			1		2	3	4	5
9	I think my parents deserve someone more successful than me.			1		2	3	4	5
10	I often ask myself whether I deserve such great parents			1		2	3	4	5
11	I feel my parents deserve more than they get from me			1		2	3	4	5
12	When my parents frustrate me, I sometimes think of running away from home			1		2	3	4	5
13	Sometimes I think my parents love me more than I deserve			1		2	3	4	5
14	Sometimes I feel I’m not good enough for my parents			1		2	3	4	5
15	Sometimes I’m angrier with my parents than with other people			1		2	3	4	5
